# Stages of Change Model: How to Deliver Nutrition Education to Adult Burn Survivors

**DOI:** 10.1016/j.cdnut.2025.104584

**Published:** 2025-03-07

**Authors:** Alyaa M Zagzoog, David Travis Thomas, Christen G Page

**Affiliations:** 1 Department of Community Health, Faculty of Applied Medical Sciences, Northern Border University, Arar, Saudi Arabia; 2 Department of Athletic Training and Clinical Nutrition, University of Kentucky, Lexington, KY, United States; 3 Department of Communication Sciences and Disorders, University of Kentucky, Lexington, KY, United States

**Keywords:** burn survivors, nutrition education, stages of change, aftercare, rehabilitation phase

## Abstract

In the early stages of recovery, adult burn survivors receive a large breadth of education that assists them as they adapt to their lives post injury. Good nutrition plays a crucial role in these early stages to improve the wellness of burn survivors. Nutrition education during the rehabilitation phase of recovery, however, is lacking for this population. Additionally, the optimum time and plan for delivering nutrition education for burn survivors during the rehabilitation phase is not yet established. Although registered dietitians (RDs) are available for nutritional interventions, they rarely provide outpatient nutrition education to burn survivors during the rehabilitation phase. The provision of a model to guide the assessment of burn survivors’ readiness to receive and act on nutrition education could assist RDs in outpatient educational efforts. This commentary article proposes a model to guide RDs in providing timely and individualized nutrition education for adult burn survivors during the rehabilitation phase. To accomplish this goal, we have modified the existing stages of change model used in clinical nutrition practice for adult burn survivors. The proposed model aims to assess the readiness of burn survivors to receive, change, and implement dietary habits during the rehabilitation phase based on two main stages: preaction and action stages. Moreover, this model proposes placement questions to assist RDs in identifying main stages, substages, and transitions between stages. It also includes four elements (four basic parts) to guide RDs while delivering nutrition education throughout each stage.

## The Role of Nutrition in Adult Burn Survivors

A burn injury is “an injury to the skin or other organic tissue primarily caused by heat or due to radiation, radioactivity, electricity, friction, or contact with chemicals” [1]. An individual who survives a burn injury is called a burn survivor. The survival rate of patients with burn injury has increased to >97% (2005–2014) because of innovations in research and medicine [2]. With medical advancements that include acute care nutrition therapy, most adults with severe burn injuries requiring burn unit admission return to their preinjury activities [3,4]. Despite these advancements, many burn survivors are often not physically or psychologically ready to adjust to their new lives, in areas such as resocialization [[5], [6], [7]]. To address this concern, there is a need to improve the physical and mental wellness of burn survivors by optimizing rehabilitation therapy after hospital discharge [5,8]. The rehabilitation phase follows the acute phase of recovery and has been described as consisting of 3 stages [9]. The first stage, the acute rehabilitation stage, starts from hospital admission to the beginning of wound healing. The second stage, the intermediate rehabilitation stage, starts from the initial grafted skin to complete wound closure. The third stage, the long-term rehabilitation stage, starts upon hospital discharge into the next life stage [9]. Although registered dietitians (RDs) are available for nutritional assessment and interventions, their primary focus in burn care has tended to be during the acute care stages; therefore, they rarely provide nutrition education to burn survivors during the rehabilitation phase in outpatient settings [10].

Sustained nutrition therapy during rehabilitation that incorporates functional foods has a potentially beneficial effect on the wellness of burn survivors. Functional foods may include a variety of nutrients that enhance health, relieve symptoms, and mitigate risk of diseases [11]. Because of this, RDs should provide burn survivors with appropriate nutrition interventions that include the provision of functional foods to enhance wellness, but most importantly, they must assess the readiness of burn survivors to receive nutrition education to support them in continued recovery. It is essential to start nutrition education with burn survivors based on their understanding of the role of food and their willingness to act on dietary behaviors to augment their physical and psychological health. Because the focus of the rehabilitation process is to facilitate wound healing and to help burn survivors return to their preinjury activities, good nutrition can promote this process. Therefore, RDs should be involved in the rehabilitation phase of recovery from burn injuries. Although several behavioral theories have been used as an intervention tool to improve health, a gap exists between current practices in nutritional education and the use of health behavioral theories, which limits the effectiveness of current nutritional practice [12,13]. RDs require a model to guide the assessment of survivors’ readiness to receive nutrition education and adopt healthy nutritional behaviors in rehabilitation settings and outpatient clinics.

## A Health Behavioral Change Model

The transtheoretical model (TTM) is an integrative theory of therapy that aims to assess an individual’s intent and readiness to change unhealthy behaviors or to implement healthy behaviors [14]. TTM is also known as the stages of change model (SCM). The SCM was developed by Prochaska and DiClemente in the late 1970s and was originally used for people who wanted to quit smoking [14]. It includes 6 stages: precontemplation, contemplation, preparation, action, maintenance, and termination. In the precontemplation stage, individuals are often not aware of unhealthy habits. Although in the contemplation stage, individuals recognize their unhealthy habits, but without the intention of changing them. In the preparation stage, individuals become intent on taking action to improve their healthy habits but have not decided how and when to start acting on their unhealthy habits. In the action stage, individuals start to act on a habit to improve their quality of life. Individuals maintain their new healthy habits in the maintenance stage [14]. Because food choices and dietary behaviors can only be modified and cannot be terminated like smoking [12,14], there are only 5 stages of the SCM when applying it to nutrition care [13].

## SCM: A Model for the Rehabilitation Phase

Progression through SCM during the rehabilitation phase for adult burn survivors depends on burn survivors’ physical and psychological/emotional behaviors. During the rehabilitation phase, survivors may experience weakness, fatigue, sleep disturbance, chronic pain, decreased muscle mass and strength, and challenges with scar management and wound care, which negatively impact their wellness [5,[15], [16], [17], [18], [19]]. Additionally, burn survivors often experience mental health challenges, such as depressive symptoms, posttraumatic stress disorder, and anxiety [18,[20], [21], [22]]. These symptoms may affect their readiness to change and implement nutritional health behaviors. The SCM provides a useful tool in addressing such reluctance. The use of the SCM helps to explain an individual’s behavior and suggests ways to achieve change through tailored intervention programs. Consequently, the SCM could be an appropriate tool for patient-centered care in outpatient clinical nutrition settings [18,22]. This article proposes a model to guide RDs in delivering timely and individualized nutrition education for survivors during the rehabilitation phase.

## SCM: A Model for Adult Burn Survivors

Adult burn survivors need long-term comprehensive care to improve their physical health and mental wellness. Nevertheless, survivors are often unaware of the importance of rehabilitation care after their injury until they later experience scar formation, physical discomforts such as pain, psychological disorders, impaired wound healing, and malnutrition [4,5,17,18]. Hence, rehabilitation and education must begin from the time of injury and continue throughout the long-term rehabilitation stage [9]. Burn survivors, depending on the severity of the burn injury, may continue to experience fatigue, weakness, and pain for ≥6–12 mo postburn [6]. The pain experience negatively affects the mental and physical health of burn survivors, which could impact their rehabilitation progress [17,18]. Therefore, the progression through the 5 stages of our proposed model is not linear—it depends on internal and external factors and may progress at different rates and stall, with a return to the previous stage when the immediate stage is unsuccessful in helping the survivors.

## SCM: A Model for Nutritional Interventions

Nutrition plays a role in the quality of life of individuals with diseases and injuries, including burn injuries. Nutrition is a fundamental element that underlies the physical aspect of the wellness model [7,15,23]. Burn survivors could benefit from nutritional interventions during the rehabilitation stage. For example, burn survivors with vitamin D deficiency have a longer wound healing time [24]. A case-control study conducted during the long-term rehabilitation phase (after 6 mo of the burn injury) found that Iranian burn survivors with >20% of total body surface area burn had significantly lower vitamin D concentrations compared with the healthy controls [25]. According to the American Burn Association Consensus Statement by Gibran et al. [26], adequate calorie and protein intake is vital for burn survivors. Additionally, the beneficial impact of glutamine supplementation has been supported by several studies, as it was shown to decrease infection rates and promote gut integrity in burn survivors [[26], [27]]. RDs should consider these factors in the nutritional plan for survivors to improve their wellness, and the proposed SCM facilitates the delivery of the plan.

## Limitations of the Existing Model

Before implementing the SCM within nutrition education for burn survivors, limitations of the existing model must be addressed. When the model was applied to patients with type 2 diabetes mellitus to assess their readiness to implement healthier dietary habits, the researchers found that some internal conditions, such as thoughts and feelings, influence the patients’ readiness for change [22]. The proposed model considers these hindering and facilitating factors to determine when and how to transition between the stages. The existing model fails to address income and social or family support, and these 2 elements are included within the external factors of the proposed model [[12], [13], [14]]. The existing model accounts for the transition between stages based on a time frame of 1–6 mo. This time frame varies among survivors due to their struggle with the consequences of burn injury for ≥6–12 mo. Therefore, the proposed model includes scored placement questions to identify the transition between substages to guide shifts among stages. Lastly, the existing model lacks clear measurements. Therefore, 4 educational elements are proposed for RDs to measure outcomes [10].

## SCM: How to Deliver Nutrition Education to Adult Burn Survivors

**FIGURE 1:**
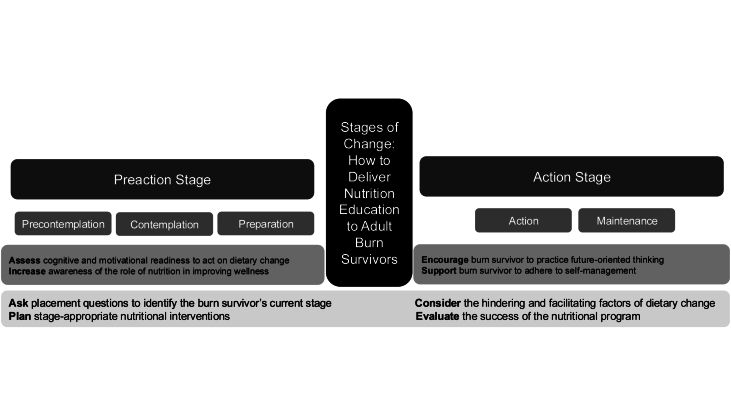
Stages of change model: how to deliver nutrition education to adult burn survivors.

On the basis of the interventions, the five stages of the existing SCM were divided into two main stages: the preaction stage and the action stage [13]. The preaction stage includes 3 substages: precontemplation, contemplation, and preparation; whereas the action stage includes two substages: action and maintenance (Figure 1). The educational processes used in the preaction stages are cognitive and motivational. Throughout the preaction substages, nutrition education should focus on minimizing barriers and increasing awareness of the perceived benefits of change to outweigh the perceived barriers. When nutritional fluency appears to increase, survivors are ready to transfer from the preaction stage into the action stage. Because a burn survivor progresses, the nutritional intervention strategy shifts to maintain the current substage or stage and enhance the movement to the subsequent substage or stage of the change process. For instance, if a survivor is not aware that they need to increase their protein intake to optimize tissue remodeling in the rehabilitation phase of the wound healing process, the nutritional intervention, in this case, is to increase awareness about the role of adequate dietary protein in increasing lean body mass (LBM) and muscle strength [27]. Therefore, this burn survivor is in the preaction stage, and RDs should work promptly to increase awareness by implementing dietary habits that aim to promote wound healing and increase LBM.

Each substage in the model has its own recommended set of interventions. Briefly, in the precontemplation stage, burn survivors are unaware of their nutritional challenges and the need to implement targeted dietary behaviors. Nutrition intervention in this first stage is to educate burn survivors about their nutritional needs. In the contemplation stage, survivors are aware of their nutritional challenges but exhibit low self-efficacy and high perceived barriers to change. Consequently, nutrition intervention at this stage involves assisting with changing or implementing healthy dietary habits. In the preparation stage, burn survivors demonstrate their willingness to implement new dietary habits. The role of RDs involves encouraging survivors to think about the steps toward their nutritional goals. In the action stage, burn survivors are ready to change and implement new dietary habits. In the action stage, survivors reflect on their competence and ability to make a change. Finally, in the maintenance stage, survivors’ nutritional behavior becomes habitual, and they plan to continue with this habit. Also, survivors adhere to self-management in the maintenance stage.

## SCM: Assessment Questions to Identify and Transition Between Stages

Prior work by Molaison [13] proposed questions to assess the readiness for dietary change in a general population. In this commentary article, assessment questions were developed and scored to fit survivors and the effort to understand their placement in the change process. The questions aim to identify patient readiness for change and where they fit within the main stages, substages, and transitions between stages of the proposed SCM. The first set of questions targets cognitive readiness, which covers the first three stages of the model, and the second set of questions targets behavioral readiness, which covers the last two stages of the model. Each question is scored one point. Placement questions can be answered with a “yes” or “no” response. The number of “yes” responses will be scored as 1 point each, whereas “no” responses will be scored as zero points. For example, if a burn survivor’s response to a question is “yes,” they will receive one point for that response. These questions provide a standardized, time- and cost-effective tool, and they do not require specific training from the RDs. Nutrition education is designed considering the scores obtained in the two main stages: the preaction stage and the action stage. Several factors could impact the placement of where a burn survivor is best aligned within the change process. These scores, however, assist with understanding survivor alignment and best fit within the change process stages and should be evaluated within the context of each individual’s circumstance. Because several internal and external factors could influence the progression of nutritional behavior change, these factors will be considered in the proposed model and in the placement questions (Table 1).

**TABLE 1:** Placement questions to identify stages and transition between stages.

Stage and substages	How to transition between stages: placement questions	When to transition: scores
Preaction stage: substages (precontemplation, contemplation, and preparation)	1. Do you think you are ready to receive nutrition education? 2. Do you think you need to make healthier dietary choices to support your burn recovery? 3. Have you increased your daily protein intake? 4. Have you considered the factors that could motivate you to start changing your diet? 5. Have you considered some possible barriers that may prevent you from making healthy changes to your diet? 6. Do you think it is important to improve your physical wellness by implementing healthy dietary behaviors? 7. Do you think it is important to improve your mental wellness by changing unhealthy dietary behaviors?	Enter the precontemplation stage if the score is ≤3
		Transition from the precontemplation stage to the contemplation stage if the score is 4 or 5
		Transition from the contemplation stage to the preparation stage if the score is 6 or 7
Action stage: substages (action and maintenance)	1. Has anything hindered you on your dietary habit change journey? 2. Did anything facilitate your success on your dietary change journey? 3. Have you looked for health resources that you may need to adhere to your new dietary habits? 4. Have you considered some sources of relapses? 5. Have you thought of any possible strategies that could be used to prevent relapse? 6. Will you continue with the new dietary change for at least a year? 7. Are you confident in sharing your new dietary change experience with your peers?	Enter the action stage if the score is ≤4
		Transition from the action stage to the maintenance stage if the score is between 5 and 7

Scoring system for the placement questions: each question in the placement is worth 1 point (score) if the response is “yes,” and a 0 if the response is “no.” In other words, the number of “yes” responses is the score.

## Factors Affecting Adult Burn Survivors’ Readiness to Change and the Transition between the Stages

Adult burn survivors likely experience internal and external factors that could hinder or facilitate their change or implementation of dietary habits. Internal factors include motivation, self-efficacy, emotions such as anxiety and depression, and the awareness of the importance of nutrition [28,29]. The external factors that could affect the commitment to change and implementation of dietary habits include extensive medical interventions (such as multiple skin graft surgeries, intubation, and infection), social or family support, income, accessibility, and affordability of food. These internal and external factors are addressed within the placement questions to assess and facilitate the readiness of burn survivors to change or implement dietary habits and the transition between stages and substages. RDs should account for these factors when providing nutrition education and intervention as part of patient-centered care [30].

## Application of the Proposed Model by RDs

The proposed model, “Stages of Change: How to Deliver Nutrition Education for Adult Burn Survivors,” can support the assessment and facilitation of the readiness of burn survivors to change and implement dietary habits during the rehabilitation phase. To implement this model, 4 basic elements should be planned by RDs while delivering nutrition education throughout each of the stages. First, it is important to ask the assessment placement questions to identify stages and the transition between stages. Second, RDs should provide nutritional intervention based on comprehensive nutritional assessment, intention, and ability to change and consider the individual context of the survivor and their circumstances. Third, RDs need to consider potential hindering and facilitating factors to prevent relapses and encourage burn survivors to practice future-oriented thinking. Fourth, RDs must evaluate the success of the nutritional program. This model provides a guide for RDs to follow when delivering nutrition education to survivors during the rehabilitation phase of recovery.

In summary, the proposed model, “Stages of Change: How to Deliver Nutrition Eductaion for Adult Burn Survivors,” provides a framework for guiding burn survivors to change their dietary habits during the rehabilitation phase.

## Author contributions

The authors’ responsibilities were as follows – AMZ: wrote the manuscript and created the table and figure; DTT and CGP: edited and reviewed the manuscript, table, and figure; and all authors: read and approved the final manuscript.

## Conflict of interest

The authors report no conflicts of interest.
